# EMDR Effects on Pursuit Eye Movements

**DOI:** 10.1371/journal.pone.0010762

**Published:** 2010-05-21

**Authors:** Zoi Kapoula, Qing Yang, Audrey Bonnet, Pauline Bourtoire, Jean Sandretto

**Affiliations:** 1 Laboratoire IRIS, CNRS, FRE 3154, Service d'Ophtalmologie-ORL-Stomatologie, Hôpital Européen Georges Pompidou, Paris, France; 2 Ecole des psychologues praticiens de l'Université Catholique, Paris, France; 3 Research Committee, EMDR Association of France, Paris, France; RAND Corporation, United States of America

## Abstract

This study aimed to objectivize the quality of smooth pursuit eye movements in a standard laboratory task before and after an Eye Movement Desensitization and Reprocessing (EMDR) session run on seven healthy volunteers. EMDR was applied on autobiographic worries causing moderate distress. The EMDR session was complete in 5 out of the 7 cases; distress measured by SUDS (Subjective Units of Discomfort Scale) decreased to a near zero value. Smooth pursuit eye movements were recorded by an Eyelink II video system before and after EMDR. For the five complete sessions, pursuit eye movement improved after their EMDR session. Notably, the number of saccade intrusions—catch-up saccades (CUS)—decreased and, reciprocally, there was an increase in the smooth components of the pursuit. Such an increase in the smoothness of the pursuit presumably reflects an improvement in the use of visual attention needed to follow the target accurately. Perhaps EMDR reduces distress thereby activating a cholinergic effect known to improve ocular pursuit.

## Introduction

### Research on EMDR treatment – role of eye movements

Eye Movement Desensitization and Reprocessing (EMDR) is a therapy which is intended to treat anxiety, stress and trauma. It was developed by Francine Shapiro [1]. EMDR is structured in 8 phases and addresses the past, present, and future aspects of the dysfunctional stored memory. The 8 phases are: history talking and treatment planning, client preparation, assessment, desensitization, installation of positive cognition, body scan, closure and re-evaluation. The client identifies an image representative of a dysfunctional target memory, rates the emotional significance of the image and identifies concomitant physiological sensations. Cognitive appraisal of the target memory is made, and the degree of currently experienced distress relative to the target memory is rated on a subjective distress scale. During desensitization, the patient returns to the disturbing image in multiple brief sets while simultaneously engaging in left-to-right eye movements in order to follow the therapist's hand movment which acts as a dual attention stimulus. Here we will emphasize that the therapist's continuous hand movement is typically done so as to stimulate the smooth pursuit eye movement physiological system. Noticeably, such movements differ from saccades which are the rapid eye movements used to refoveate targets presented at distinct positions. The pursuit system is activated by the velocity of the continuously moving target while the saccade system is stimulated by discrete position information i.e., the location of a target at different positions. The neurophysiology of the pursuit oculomotor system will be presented later. Returning to the EMDR protocol, after each set the patient is asked what associative information was elicited during the procedure.

EMDR stimulated various lines of research including laboratory research on the mechanisms involved and on the physiological correlates of EMDR. In what follows we will briefly review some of these studies.

Van den Hout et al. [2] used a laboratory experiment to test the role of eye movements on vividness and emotional rating of personal memories. Healthy volunteers recalled positive or negative memories. Memories were recalled while participants either performed rapid eye movements (following the experimenter's hand movement as in EMDR) or finger tapping or simply refrained from performing any dual task whatsoever. Negative memories became less negative and positive memories became less positive following the eye movements, in contradiction from other interventions which failed to produce a similar effect. Subsequently, when participants were asked to recall the event and rate its vividness and emotionality, an eye movement effect was found again. Thus, eye movements also influence future recollections after the EMDR session. According to the authors, the after effect is not compatible with the visuospatial sketchpad theory proposed earlier by Andrade et al. [3] according to which eye movements render the target image less vivid thus reducing emotionality.

Lee & Drummond [4] investigated the effectiveness of the eye movement component compared with mere instructions in students who were asked to recall distressing memories. EMDR treatment with eye movements or an identical procedure with eyes stationary was also applied. In addition, therapists encouraged participants either to assume a distanced perspective vis-à-vis the traumatic memory or to maximize on reliving the experience as is done in exposure treatments. The results indicate both a significant reduction in distress for the eye movement condition as well as a significant reduction in the vividness of the memory after the eye movements were combined with distancing instructions. In contrast, eye movements combined with “reliving” instructions did not reduce vividness ratings. Thus eye movements appear to facilitate the processing of distressing memories. The authors conclude that the mechanism of change in EMDR is not the same as in traditional exposure experiments and point out the potential interest of distancing instructions as part of the EMDR protocol.

However, the objectives of the present study are perhaps more congruous with studies centered squarely on the various physiologic correlates of EMDR. Barrowcliff et al. [5] examined different hypotheses concerning the orienting response as a possible mechanism in the EMDR treatment in a laboratory experiment. Subjects were confronted with auditory stimuli of different intensity while engaging in either eye movements or fixation (stationary eyes). Electrodermal responses were measured. Skin conductance responses were higher with eyes stationary indicating higher magnitude of orientating responses. The authors conclude that eye movements diminish the arousal caused by auditory stimuli. In a second study attention tasks of high or low demand were coupled with auditory stimuli; electrodermal responses showed higher amplitudes with high attention demand. Thus the effect of eye movements in the first experiment could not be attributed to attention alone. Extrapolating to hypothetical mechanisms underlying EMDR, the authors favour the de-arousal hypothesis [6] rather than the intensified orienting reaction hypothesis proposed by Armstrong & Vaughan [7], or the non-orienting reflex centered hypothesis as sustained by Wilson et al. [8].

Barrowcliff et al. [9]examined the role of eye movements on subjective and psychophysiological measures of arousal and distress associated with positive or negative memories. Eye movements compared to eyes stationary condition reduced vividness and emotional valence for both positive and negative memories. Importantly, reduction of electrodermal arousal was observed only in the eye movement condition and only with respect to negative memories. The authors propose that the presence of perceived threat for negative memories is essential in order for such de-arousal to occur given that de-arousal follows activation of a negative visceral loop, as suggested by MacCulloch & Feldman [6]. The effects of eye movements on positive memories do not support the hypothesis of eye movements in EMDR as constituting a “reassurance reflex”. Thus the authors propose that two mechanisms are operating in parallel: disruption of the visuospatial sketchpad (mentioned above) and the process of physiological de-arousal.

More recently, Elofsson et al.[10] examined heart rate, respiration, fingertip temperature and skin conductance on patients who had undergone EMDR-treatment following Shapiro's protocol. This study directly assess physiological changes across real life EMDR treatment sessions. Eye movements caused a shift in the autonomic balance as indicated by a decrease in heart rate and skin conductance as well as an increase in finger temperature. Breathing frequency and oxygen saturation also decreased. The authors conclude that EMDR activates a cholinergic response and inhibits sympathetic systems. They discuss several explanatory hypotheses of EMDR action (distraction, conditioning, orienting response activation and REM-like mechanisms). They suggest that the physiological reactivity observed has similarities with the REM-sleep pattern. Another study from Sack et al. [11] also has the merit of examining psycho-physiological correlates of EMDR during real life treatment sessions. The results show significant de-arousal in terms of a decrease in heart rate and breathing rate and an increase in parasympathetic tone when comparing the first two and the last two sets of the desensitization procedure. The authors conclude that redirecting the focus at each set elicits an orienting response with psycho-physiological de-arousal. They also highlight the difficulty in determining whether or not these results stem from single or multiple orienting responses elicited during stimulation and directed toward the waving hand of the therapist. In the same vein, Aubert-Khalfa et al. [12] measured heart rate and skin conductance in six patients with post-traumatic stress disorder (PTSD) before and after an EMDR session under two conditions: 1) the subject was in a relaxed state 2) the subject was visualizing his or her traumatic event. A significant reduction of the symptoms was observed. Moreover, after only one EMDR session heart rate and skin conductance during the trauma recall condition decreased significantly compared to the relaxed state condition.

Several brain imaging studies have also been conducted to assess modifications in cerebral activity following EMDR treatment, some of them are briefly presented below. Levin et al. [13] assessed brain activation with Single Photon Emission Computed Tomography (SPECT) prior to and after 3 sessions of EMDR treatment. Recall of the traumatic event following EMDR increased activation of the anterior cingulated gyrus and of the left frontal lobe. The authors suggest that treatment of PTSD by EMDR does not reduce arousal at the limbic level, but instead enhances the ability to differentiate a real from an imagined threat. Lansing et al.[14] also used SPECT before and after EMDR treatment in police officers with PTSD. They reported a reduction of PTSD symptoms after EMDR treatment which was accompanied by decreased activation of the occipital, left parietal and right precentral frontal lobes, as well as a significant increase in the activation of the left inferior frontal gyrus. A more recent study [15] examined train drivers suffering from PTSD after experiencing train accidents. SPECT was performed before and after EMDR therapy while they listened to a script portraying the traumatic event. Results were compared to those of a control group which had experienced similar train accidents without developing PTSD. Between the two groups significant differences were found in the orbitofrontal cortex and the temporal pole before and after treatment. For patients with PTSD who responded to EMDR treatment significant differences before and after EMDR were also observed in the frontal, parieto-occipital and visual cortex and in the hippocampus. Compared with the control group, the significant difference observed before EMDR disappeared after EMDR treatment.

To summarize, research on mechanisms and physiological correlates of the EMDR indicates the importance of eye movements in EMDR treatment. The controversy over the mechanisms behind the workings of EMDR, is one of the main issues of ongoing research. Yet, it may well be that the above mentioned hypotheses are equally true given that EMDR treatment is *syncletic* and integrative, i.e., it presumably mobilizes several mechanisms. There is no doubt that eye movements are important key point of this method. Yet, to the best of our knowledge, no study exist with physiological recording and analysis of the eye movements *per se*. Electrophysiological studies, patient and brain imaging studies allowed to identify the cortical–subcortical neural circuitry subtending the programming and execution of different types of eye movements. Thus, oculomotor systems are among the best understood sensorimotor systems in the primate brain; they constitute a model for testing motor control theories for exploring brain function and plasticity. Our study introduces a physiological examination of the eye movements *per se* combined with EMDR sessions. As the movement elicited by the waving hand of the therapist in Shapiro's protocol is typically a smooth pursuit eye movement we undertook a physiologic study of this movement. Another reason for studying pursuit, is that among the different types of eye movements (e.g. saccades, pursuit etc.) it is pursuit eye movement which is most studied in the field of psychiatry and psychopathology. Next we will present briefly some studies relevant to this goal.

The motivation of the present study comes also from clinical observation of changes in smoothness of pursuit eye movements during EMDR session. We hypothesize that lack of smoothness of pursuit eye movements reflects stress and emotional perturbation, and that reduction of the above by EMDR will increase such smoothness. In other words, we expect a similar effect as that shown in physiological studies, e.g. effects of nicotine on pursuit in schizophrenia. These studies will be reviewed below after a brief presentation of physiological aspects of pursuit eye movements.

### Physiological aspects of pursuit eye movements

Pursuit eye movement is the ability of the eyes to smoothly follow a small object moving in a stable environment as mentioned. The stimulus for the initiation of smooth pursuit is the velocity of the moving object, i.e. the slippage of the image away from the fovea. Processing of visual motion is primarily generated in the medial temporal lobe (area V5, e.g.[16], [17]. As reviewed by Krauzlis [18] pursuit is not a simple automatic behavior in which visual motion signals from visual areas are transferred to motor regions in the cerebellum to produce motor commands. Pursuit involves an extended cortical-sub-cortical network including the frontal eye fields, parietal areas, basal ganglia, superior colliculus, cerebellum and nuclei in the brainstem. Krauzlis [18] emphasized the similarity of the pursuit network with that of saccades sharing the same sensory motor function. After the initiation of the pursuit, the eyes follow the target using both retinal and extra retinal information to maintain the image in the fovea: *the predictive pursuit* is believed to be mediated by a network consisting of the medial superior temporal lobe, the posterior parietal cortex, the frontal eye field, and the cerebellum [17], [19], [20].

Pursuit may not be perfect even in healthy subjects. Several types of deficits may exist: slow initiation, low gain associated with saccade intrusions, or predictive saccades. Here we will be interested mainly in one type of small saccades appearing during smooth pursuit (SP), i.e. catch-up saccades (CUS). Catch-up saccades occur when the eyes are lagging behind with lower velocity than that of the moving target. Although the mechanisms that control both CUS and SP are still poorly understood, common structures seem to be involved in their control. For example, error signals on motion or position of the moving target in the superior colliculus (SC) could be shared by the saccadic and smooth system [21]. Lesions of the oculomotor cerebellar vermis affect both saccades and smooth pursuit [22]. At the cortical level, there is anatomical evidence for connections between structures containing subregions for saccades and pursuit [23].

### Pursuit abnormalities in psychopathology

Several studies reported pursuit deficits in psychological disorders such as *schizophrenia* or *autism*. Abnormalities during SP common in both schizophrenic patients and their relatives were reported by Ross et al.[24]): schizophrenic patients showed low gain and increased rates of CUS [24], [25]; other studies reported trouble in pursuing very fast targets, namely low pursuit gain and poor initial acceleration compared to control subjects [26]. This impairment is negatively correlated with activation in areas known to play a role in pursuit, such as the frontal eye field [26]. For a recent review on pursuit abnormality in schizophrenia see Rommelse et al. [27].

Sherr et al. [28] examined the effects of acute administration of nicotine on measures of pursuit eye movements and visual attention. Nicotine significantly improved pursuit gain for target velocity of 18.7°/sec, while no differences were observed for visually guided saccades or visual attention. Such effects were observed in schizophrenia patients only, suggesting abnormality in neuronal nicotine system. Tregellas et al. [29] examined effects of nicotine during SP eye movement task in schizophrenia. They reported nicotine-associated decrease of activity in the parietal eye fields that are consistent with improvement in inhibiting function thereby reducing saccade intrusions during pursuit. The authors also reported less activity in the hippocampus but higher activity in the anterior cingulated, posterior cingulated, precuneus and MT/V5 areas; enhancement of activity in such brain regions involved in attending to moving stimuli could be the other mechanism by which nicotine improved pursuit performance in schizophrenia.

Patients with autism also show low pursuit gain for its initial period of 100 ms, but these deficits were only seen when targets moved from the center into the right visual field [30]. Note that patients with autism have also difficulties with various types of saccades: anti-saccades (voluntary saccades directed opposite to a visual target), memory-guided saccades (saccades to a target previously flashed), predictive saccades, more reflexive saccades such as those obtained when the central fixation point switches off before peripheral target presentation (gap task) [31]. Abnormalities of pursuit were also reported in dyslexia. Black et al. [32] reported high rates of CUS during smooth pursuit in dyslexics. Eden et al. [33] found poor smooth pursuit in dyslexics, particularly when pursuing a target moving from left to right.

This review although not exhaustive indicates abnormalities of pursuit eye movements in several disorders. Whether abnormalities of pursuit can be a biological marker of psychological disorders is a controversial issue [34]. Some abnormalities of pursuit may be due to attention fluctuation rather than to deficit of the cortical-subcotical circuits involved in the generation of such movement. Indeed, increased rates of CUS occur also in healthy subjects in double tasks requiring division of attention such as listening and tracking a target visually [35]. We can conclude, however, that quality of pursuit eye movements, particularly their smoothness, can reflect brain function, cognition and attention deployment. The above cited studies are particularly relevant as they provide a context for the interpretation of eventual EMDR effects on the physiology of pursuit eye movements (see Discussion).

The goal of the present study was to objectivize in healthy volunteers the effects of an EMDR session on the physiology of pursuit eye movements studied in a laboratory setup. Pursuit eye movements were measured with video-oculography before and after an EMDR session carried out with persons evoking emotive memories. The frequency of CUS and the gain of smooth components during pursuit were analyzed. The results show decrease of frequency of CUS and increase of gain of smooth components after EMDR but only when the session was complete and distress dropped to zero.

## Methods

### Ethics Statement

The eye movement investigation adhered to the tenets of the Declaration of Helsinki and was approved by the local human experimentation committee, CPP Il de France II (No: 07035), Hospital Necker in Paris. Consent was obtained from all subjects after the nature of the procedure had been explained.

### Subjects

Seven healthy subjects (female, 32.2±8.5 years, mean ± standard deviation) participated in the study. Three of them were students in the psychology university and the others were psychotherapists; all of them had intellectual interest for the EMDR relative to their professional projects. They were questioned on known physical, psychiatric illness, medications, prior or current followed up by psychotherapist; ophthalmologic problems and pregnancy were also excluded. All subjects were healthy, well functioning with stable everyday life. Moreover, they were invited to choose an autobiographic negative event of moderate distress; this was evaluated afterwards following the EMDR protocol (see below). General information about EMDR was given as required by the EMDR protocol. Information about the pursuit physiologic eye movement test was also given: presentation of the task, of the viseo-oculography apparatus, and of the purpose of the test - understanding the neuro-physiology of pursuit eye movement control in humans. To avoid bias, the specific hypothesis of the study, i.e. possible influence of the EMDR on parameters of pursuit eye movements, was not communicated to the subjects. The EMDR therapy was done by a therapist who had received a training (level 2) accredited by the French EMDR association.

### EMDR and Measures

EMDR protocol involves evaluation of distress intensity. In this study, the Subjective Units of Discomfort Scale was used [36]. This is one of the most widely used measures of intensity of subjective distress. It is an 11-point scale where 10 reflect the highest level of distress or disturbance and 0 the lowest level or absence of distress/disturbance. It has been shown to correlate with several physiological measures of stress [37]. The therapist followed Shapiros' [1], [38] recommendations for targeting on old memory. Table 1 shows the SUD scores before and after the session indicating a discomfort of moderate degree. The VOC (validity of cognitive scale) indicating the person-self cognition is also shown before and after except when the session was not complete [1], [4], [38].

**Table 1:** SUDs and VOC before and after EMDR.

	Age (Year)	Gender	SUDs Before	After	VOC Before	After
S1	22	F	6	0	3	**7**
S2	24	F	6	2.5	2	
S3	30	F	8	3	1	
S4	42	F	4	0	2	**7**
S5	38	F	6	0	4	**7**
S6	38	F	7	0	2	**7**
S7	38	F	8	0	3	**7**
Mean (SD)	33(7.8)		6.4 (1.4)	0.8 (1.4)	2.4 (1.0)	7 (0)

### Oculomotor task

**Figure 1:**
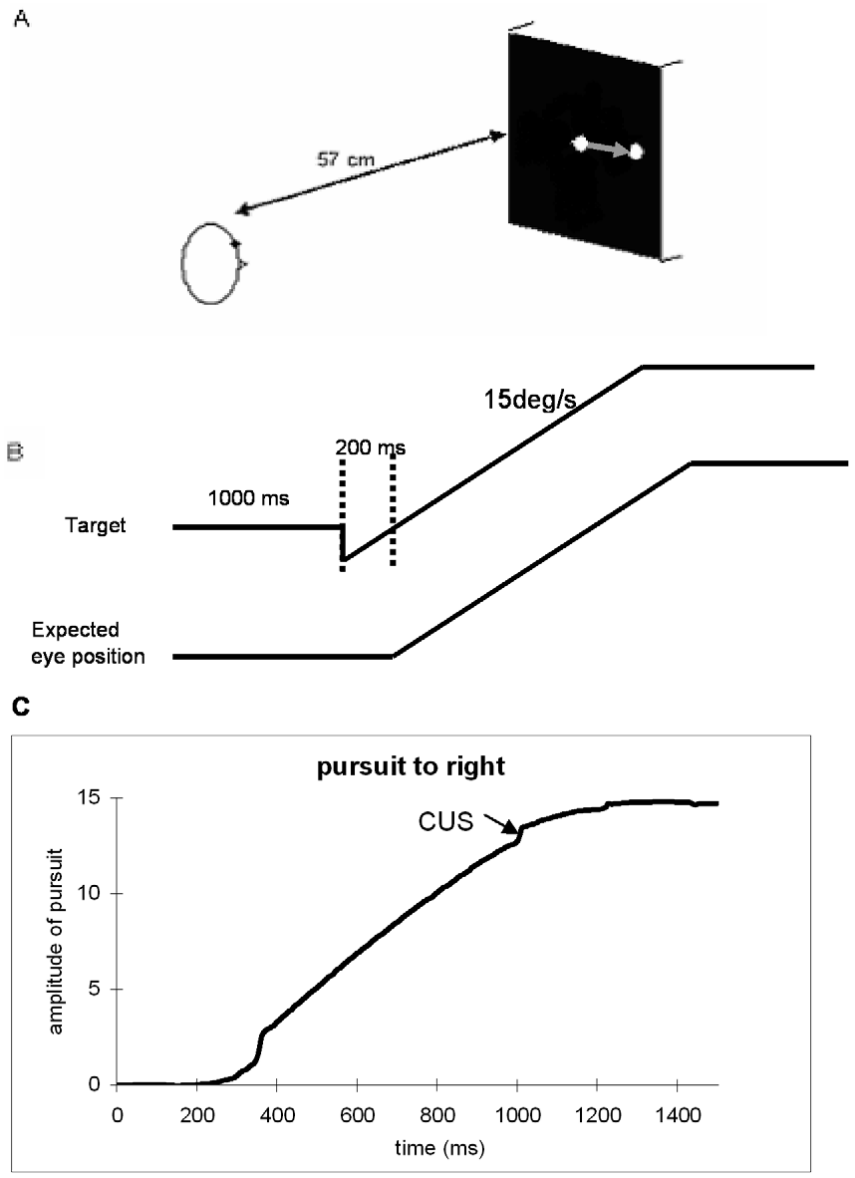
The smooth pursuit task. (A) Spatial arrangement for smooth pursuit: one white dot on the black computer screen, 57 cm from subject's eyes, moving from center to right or left (15°), randomly. (b) Temporal arrangement: the white dot moves to opposite direction to the pursuit then returns to center (200 ms) to begin pursuit, the stimulus at velocity of 15°/s. (c) Typical recording of normal rightward pursuit is obtained by averaging the position signal of the two eyes (LE+RE)/2; CUS indicates catch-up saccades during the pursuit.

The stimulus consisted of a black dot (0.2°) in the center of a grey background on the PC screen (Figure 1A). Each trial started with a fixation period of 1 s at the position of center of the screen. The dot first stepped by 2° to the left or right of fixation dot and then moved in opposite direction (Figure 1B). This step reduced the probability of occurrence of the first catch-up saccade during pursuit initiation [39]. The dot velocity was always 15°/s and it stopped when an eccentric position of 15° was reached. Each block included 30 trials randomly interleaved (15 to left and 15 to right) lasting about 4 minutes. The instruction given to the subject was to pursue the moving dot as accurately as possible. The task was repeated before and after the EMDR session. Before the pursuit a calibration task was run during which the subject followed a dot that stepped from center to left, right, up, down at 15°, returning each time to the center; the task elicits saccades.

As the goal of the study was to test for lasting physiologic effects after the EMDR on pursuit eye movement no instruction was given to connect with the target event worked during the EMDR session.

### Eye movement recording

The Eyelink II video eye tracker was used; it consisted of infra-red cameras (CMOS sensors). The sampling frequency was 250 Hz.

### Data analysis

Eye movement signals were calibrated using a linear function with factors extracted from the calibration task. We measured the gain of pursuit, e.g. ratio of mean velocity of pursuit phase (without saccades) to the stimulus velocity; the number of catch-up saccades (CUS, see Fig. 1C) during pursuit and their mean amplitude. As in other studies [40], [41] CUS were defined as saccades occurring during pursuit, in the direction of dot motion, which take the eyes from a position behind the target to one nearer the target. Saccades that occurred after the start of dot motion but prior to the start of pursuit were omitted. Saccades were identified on the basis of velocity and acceleration criteria (eye velocity >35°/s; eye acceleration >1000°/s^2^; similar criteria have been used by others [40].

The Wilcoxon signed rank test (software Statistica) was used for group comparisons before and after EMDR measurements.

## Results

### EMDR subjective measures

Table 1 summarises the results of EMDR. Prior to EMDR session the average SUD value was 6.43; after the session this value dropped to 0.79. Note that for two subjects (S2, S3) the values remained >2 and these sessions were not complete. Reciprocally, the group mean VOC was low before the EMDR session (2.43) and increased to 7 after the session. No VOC was measured for the subjects with incomplete sessions.

### Eye movement measures

#### Qualitative results

**Figure 2:**
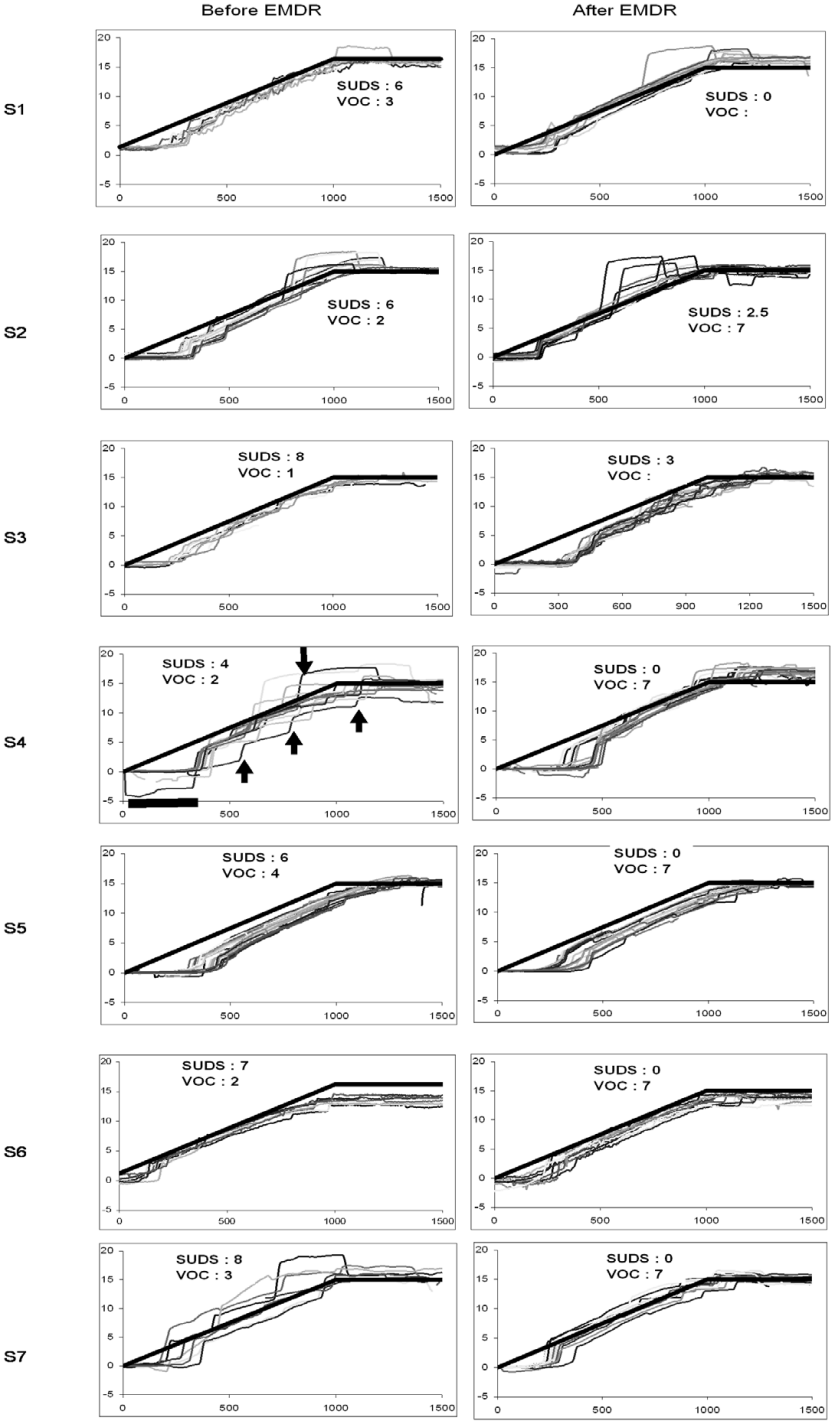
Eye movements recorded during the pursuit task. Individual traces of rightward smooth pursuit eye movements before and after EMDR for each subject. Each trace shows the instantaneous eye position over time during an individual trial. The thick diagonal line shows target displacement from centre the right of the screen. The eye movement starts after a latency period (see thick horizontal segment, s4). The eye position lagging behind target position frequently small catch up saccades are made (upward arrows). Occasionally the eye anticipates, e.g. going beyond target position and then a small saccade returns it back on the target (see downward arrow). The values of SUD and VOC are shown in cells of each figure. After EMDR all subjects show decrease of SUD and increase of VOC (when measured).

**Figure 3:**
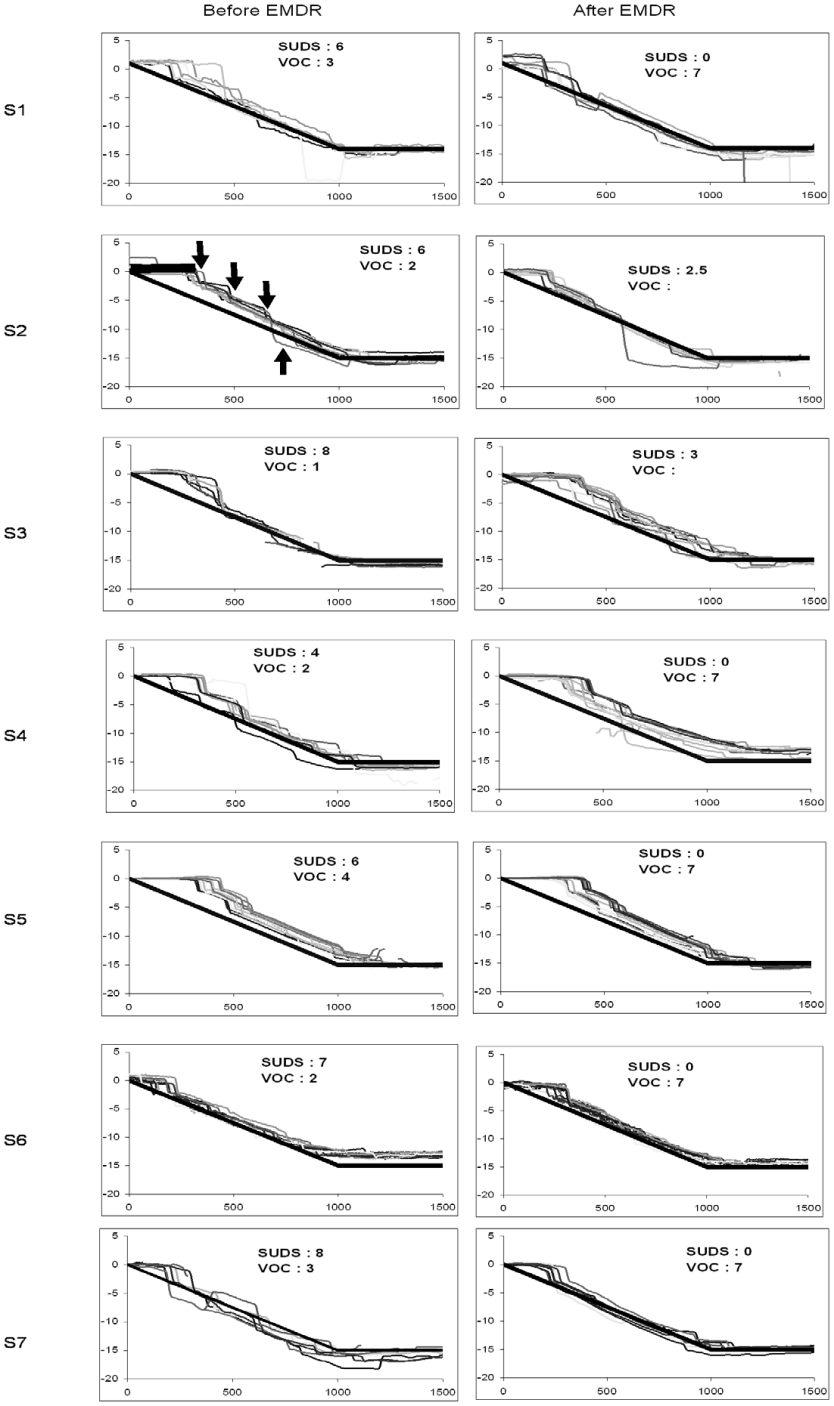
Eye movements recorded during the pursuit task. Individual traces of leftward smooth pursuit eye movements before and after EMDR for each subject. Each trace shows the instantaneous eye position over time during an individual trial. The thick diagonal line shows target displacement from centre to the left of the screen. The eye movement starts after a latency period (thick horizontal segment, s2). The eye position lagging behind target position frequently small catch up saccades are made (downward arrows). Occasionally the eye anticipates, going beyond target position and returns back (see upward arrow). All other notations as in Fig. 2.

Figure 2 and 3 show superimposed trajectories of pursuit eye movements before and after the EMDR session for rightward and leftward target moving trials, respectively. Each trace is the instantaneous eye position (in degrees) over time while following the moving dot on the PC screen. The dot moved smoothly with a velocity of 15°/s and its movement starts at time zero. The eye starts moving after a certain period of time, the so called latency period (indicated by the thick horizontal segment). The thick oblique line indicates the position of the target dot over time. If pursuit eye movements were perfect they should be straight lines superimposed to the target line. This is not the case. The eye position in most cases lags behind the target and small saccades were made to catch up (indicated by upward arrows in Fig. 2 and downward arrows in Fig. 3). Instances of anticipatory saccades are indicated by downward arrows in Fig. 2, upward arrows in Fig. 3. As anticipatory saccades drive the eye beyond target position, another small return saccade occurs. The majority of saccade intrusions are catch-up saccades. Otherwise, all subjects followed the dot target until it reached the stop point at eccentricity of 15 degrees; but their pursuit was frequently interrupted by saccades, particularly prior to the EMDR session. The SUD and VOC for each subject are also shown. Qualitative inspection of the traces together with the SUD and VOC values shows improvement of smoothness, e.g. less saccade intrusions for S1, S4, S6, S7. No noticeable changes could be observed for S2 and S3 whose SUD remained high after EMDR (incomplete EMDR sessions); particularly subject S3 having highest SUD and lower VOC prior to the EMDR session and persisting high SUD after the EMDR session, her results were not included in the statistical group analysis (see below).

#### Quantitative results

**Figure 4:**
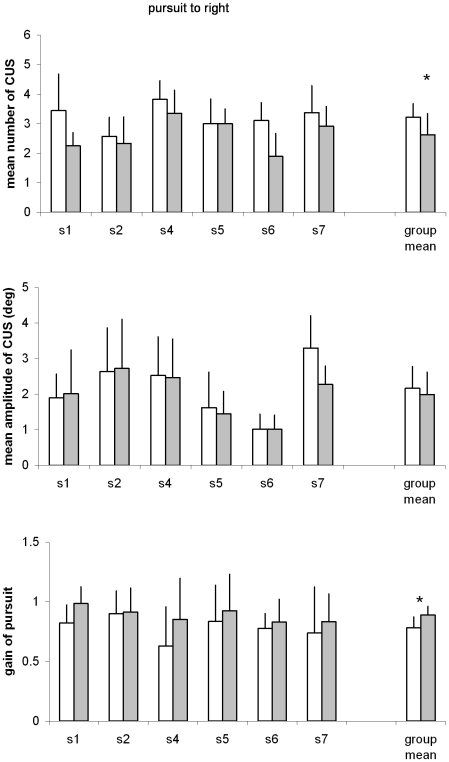
Parameters of pursuit eye movements. Individual mean values with their standard deviation of number of CUS (A), amplitude of CUS (B) and gain of pursuit (C); data are shown before and after EMDR for smooth pursuit to right. Group mean values are presented on the right of each group. Asterisks show statistically significant difference.

**Figure 5:**
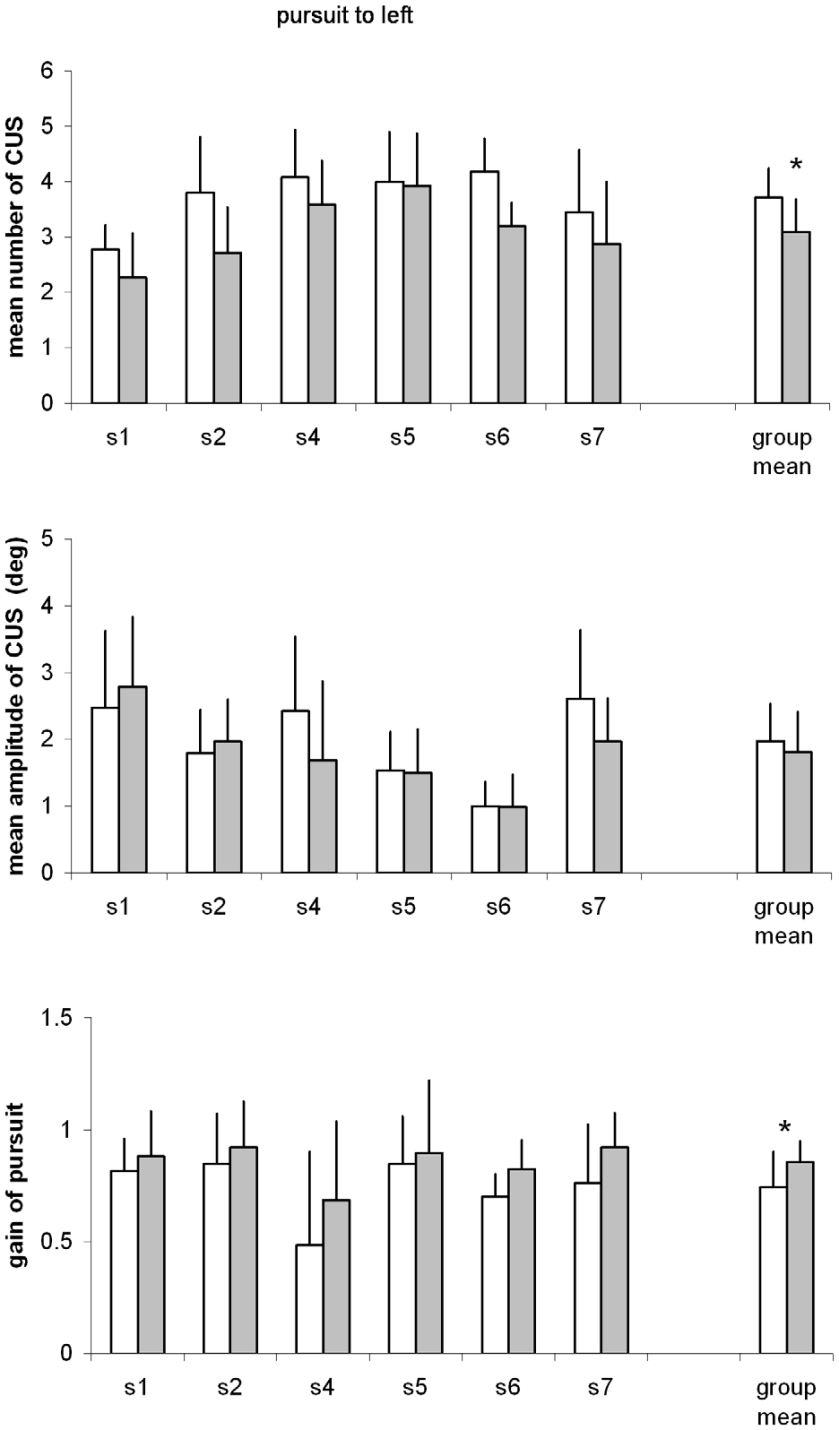
Parameters of pursuit eye movements. Individual mean values with their standard deviation of number of CUS (A), amplitude of CUS (B) and gain of pursuit (C); data are shown before and after EMDR for smooth pursuit to left. All other notations as in Fig. 4.

Figures 4 and 5 present mean number and mean amplitude of CUS, and the gain of smooth pursuit to right and to left, respectively. The Wilcoxon tests applied to the average number of CUS showed statistically significant decrease after EMDR for both directions (both T = 1, p<0.05); reciprocally, the gain of smooth pursuit component showed statistically significant increase after EMDR for both directions (T = 0, p<0.05). The mean of amplitude of CUS did not change significantly after EMDR. Inspection of individual results in Figures 4 and 5 shows that these effects are systematic concerning almost all subjects.

### Comparison CUS with controls

The question arises whether the amount of CUS prior to EMDR session was within the normal range for these subjects. We extracted normal values from the literature, e.g. Friedman et al. [41]. Also we recorded pursuit from 2 healthy subjects in our laboratory using the same setup as for the subjects of the EMDR; their results confirmed the rates of CUS and the gain of smooth pursuit reported in the study of Friedman et al. (1992): 0.85±0.30 (mean/SD) CUS per second; the mean gain of smooth pursuit component was 0.93±0.05. Comparisons of each subject's data with normal values show higher rate of CUS for all our subjects, particularly prior to the EMDR session; their values being two standard deviations from the normal values, except for subject 2. This was the case for both, CUS and gain of smooth pursuit components. In contrast, the size of the CUS for our subjects was no different from normal values.

### Anticipatory saccades and Square wave jerks

For other types of saccade intrusions, such as anticipatory saccade or square wave jerks (SWJ, see Methods), no EMDR effect was found. In effect, the group mean rates of these saccades remained small and similar before and after the EMDR (excluding subject S3). Before EMDR anticipatory saccades occurred at rates of 4.8 times/minute, SWJ 4.2 times/minute; after the EMDR the values were 3 times/minute, and 3.6 times/minute, respectively.

## Discussion

The study aimed to objectivize the quality of smooth pursuit eye movements in a standard laboratory task before and after EMDR session. The EMDR session was applied on healthy subjects and on autobiographic worries causing moderate distress. The EMDR session was complete in 5 out of the 7 cases, and distress measured by SUD decreased to near zero value. Pursuit eye movements improved after the EMDR session, namely the number of CUS decreased and reciprocally, the gain of the smooth components of the pursuit increased. So the overall effect of EMDR was an improvement of the smoothness of the pursuit, i.e. as the eyes were lagging less behind the target there was less need for CUS. Such improvement presumably reflects better employment of visual attention resources needed to follow the moving target. Indeed prior studies have shown that the rate of CUS can increase in dual tasks in which subjects have to follow the pursuit target while listening to a voice pronouncing a letter series [35]). Importantly the rates of CUS prior to EMDR for all subjects were abnormally high. Thus, EMDR normalized pursuit eye movement behaviour in such subjects. The improvement of the pursuit could reflect a lasting benefit in the subject's capacity to use better her visual attention resources.

One could also argue that the improvement is due to practice. To our knowledge, there is no evidence for decrease of rates of CUS just by practice. Analysis of our data over time, e.g. comparison between the first and last pursuit trial in a given session does not show a trend for such reduction. Note also that the time interval between before and after EMDR eye movement recording was about 90 min. A fatigue effect would cause increased rates of CUS but this was not the case. Rather, we suggest that improvements of pursuit could be related to modifications of cerebral activity due to decrease of distress after the EMDR session. This is in line with other studies; for instance with the study of Tregellas et al. [29] on nicotine effects in schizophrenia who reported decrease of CUS during pursuit that were associated with decrease of activity of the parietal eye fields. Prior studies from our team have shown that the posterior parietal cortex is greatly involved in the generation of many types of eye movements (saccades but also vergence, i.e. convergent or divergent movements of eyes allowing to adjust the angle of optic axes to different depths)[42], [43]. Namely this region is involved in the reflexive initiation of all types of eye movements; interference by transcranial magnetic stimulation (TMS) caused latency prolongation for all eye movements, and this is compatible with models attributing to this area a major role for both visual attention and eye movement control. [44]. Brain studies also showed increased cerebral activity in areas such as the anterior and posterior cingulate gyri, and precuneus that are likely to be involved in attentive target tracking leading to smooth ocular pursuit eye movements [29], [45], [46].

Perhaps EMDR reducing distress activates a cholinergic effect improving pursuit eye movements as does nicotine in schizophrenia [47], [48]. This interpretation is also in line with physiologic correlates of EMDR reviewed in the Introduction. For instance, it is compatible with the study of Elofsson et al. [10] who recorded many correlates (finger temperature, heart rate, skin conductance, blood pulse oximeter oxygen saturation) and observed modifications after EMDR compatible with activation of the cholinergic and inhibition of the sympathetic systems. EMDR presumably drops arousal and stress, and increases attention. It should be emphasized that we observed such positive effects only for subjects with complete EMDR sessions. The two subjects for whom the SUD remained high showed no significant modification of their pursuit eye movements. This is an indication that increased smoothness of pursuit after EMDR is related to positive EMDR effects. Future studies with more subjects are of interest to search for correlation between EMDR results and smoothness of pursuit. Perhaps pursuit smoothness can be a subtle neurophysiological marker of the efficacy of EMDR treatment.

In conclusion it should be emphasized that the effects of pursuit improvement reported here are present after the EMDR session, and while the subject is in a laboratory setup. This suggests lasting beneficial effects. Eye movement semiology is known to be a great tool for exploring brain function and plasticity [49]. This preliminary study might be a starting point for further studying in this field with other types of eye movements bringing together neuroscience and psychotherapy. Some suggestions for future studies follow. First, objective recording of eye movements during the EMDR itself together with recording of the waving hand of therapist would be of interest. This should allow to assess objectively the quality of eye movements over successive sets of the desensitization procedure, and to correlate with instances of abreaction, blockage etc. Our clinical observation is that important changes occur in the physiology of eye movements within the desensitization period from one set to the next. Remote eye trackers (eye video camera placed at distance) could have the advantage in recording person's eye movements under real life EMDR sessions. It is also important to follow up treatment over many EMDR sessions combining recordings of eyes of the patient, therapist's hand, SUD and VOC measures. Another interesting avenue is to cross-correlate effects on eye movement with those on other physiologic parameters (e.g. heart rate, skin conductance also recorded simultaneously). Such data may provide interesting information testing further specific hypotheses for mechanisms of EMDR action. For instance, the smoother the eye movement pursuit is the most powerful the visuospatial sketchpad mechanism should be. Eye movement studies combined with brain imaging studies before and after, or even during real life EMDR sessions are of also of major interest. Finally, it is also important to establish whether the EMDR effect reported here is specific to smooth pursuit eye movements (which are the movements stimulated by the therapists moving hand) or if other types of eye movements are also boosted. As the EMDR involves distancing from the target memory it would be of interest to test possible effects on vergence eye movements that regulate the angle of optic axes according to depth and which attention and fixation are focused. Our research team aims to apply further our expertise on eye movements in the EMDR field.
